# Smoking and Alcohol During Pregnancy: Effects on Fetal and Neonatal Health—A Pilot Study

**DOI:** 10.3390/jcm14197023

**Published:** 2025-10-03

**Authors:** Martina Derme, Marco Fiore, Maria Grazia Piccioni, Marika Denotti, Valentina D’Ambrosio, Silvia Francati, Ilenia Mappa, Giuseppe Rizzo

**Affiliations:** 1 Department of Maternal and Child Health and Urology, Policlinico Umberto I, Sapienza University of Rome, 00161 Rome, Italy; mariagrazia.piccioni@uniroma1.it (M.G.P.); marika.denotti@uniroma1.it (M.D.); v.dambrosio@policlinicoumberto1.it (V.D.); mappa.ile@gmail.com (I.M.); giuseppe.rizzo@uniroma1.it (G.R.); 2 Institute of Biochemistry and Cell Biology—National Research Council, Department of Sensory Organs, Sapienza University, 00161 Rome, Italy; marco.fiore@cnr.it; 3 Department of Experimental Medicine, Sapienza University of Rome, 00161 Rome, Italy; silvia.francati@uniroma1.it

**Keywords:** smoking, alcohol, pregnancy, fetal growth restriction, oxidative stress

## Abstract

**Background/Objectives**: Alcohol and smoking during pregnancy may be associated with several complications, but the underlying mechanism is still unclear. The aim of this study was to evaluate the role of oxidative stress induced by smoking and alcohol during pregnancy and their effects on fetal and neonatal outcomes. **Material and methods**: We considered pregnant women at term. Validated questionnaires were used to investigate smoking and alcohol habits. Ultrasound was performed to evaluate fetal weight, amniotic fluid index, and maternal-fetal Doppler velocimetry. At the time of delivery, we collected a tuft of maternal hair, maternal venous blood, and cord blood. In these samplings we determined in phase I nicotine, cotinine, and ethyl glucuronide on the maternal keratin matrix with the gas chromatography-mass spectrometry technique. In phase II, the Free Oxygen Radicals Test (FORT) and Free Oxygen Radical Defense (FORD) test were used to assess circulating reactive oxygen species (ROS). **Results**: 119 pregnant patients were enrolled (*n* = 62 for smoking and *n* = 57 for alcohol). Twenty-six patients (42%) out of 62 were active smokers. Three patients (5%) out of 57 were alcoholic consumers. Mean neonatal weight and mean placental weight were significantly lower for active smokers (*p* = 0.0001). The neonatal weight was in the 1st–2nd percentile for all alcohol abusers. Considering two subgroups (*n* = 10 non-smokers and *n* = 10 smokers) for ROS determination, a statistically significant higher oxidative stress in the blood of smoking patients was evidenced (*p* < 0.0001). In cord blood the differences were not statistically significant (*p* = 0.2216). **Conclusions**: Fetal growth restriction was present in the group of active smokers and in patients with alcohol abuse. Oxidative stress was higher in smoking patients than in non-smokers. However, in cord blood, FORT was negative in all cases, suggesting a protective mechanism in utero. Given the limited sample size, the results obtained are preliminary and require future studies.

## 1. Introduction

According to the recent WHO global report, 65.5% of childbearing women in European countries consume alcohol, and given that almost half of pregnancies are unplanned (42%), the risk of alcohol consumption during the early stages of gestation is very high. The European Region exhibits the highest prevalence of alcohol consumption during pregnancy, averaging at 25%, followed by the American Region (11.2%), the Western Pacific Region (8.6%), the African Region (10.0%), and the Southeast Asia Region (1.8%). The lowest prevalence is present in the Eastern Mediterranean Region (0.2%) [1].

Fetal Alcohol Spectrum Disorders (FASD) is an umbrella term that encompasses a range of conditions caused by prenatal alcohol exposure (PAE). FASD is a global health concern, with prevalence rates ranging from 1% to 5% in certain high-risk populations.

Globally, 52.9% of women who smoked daily continued to smoke daily during pregnancy. The European Region had an estimated prevalence of smoking during pregnancy of 8.1% [2]. Globally, 72.5% of women who smoked during pregnancy were daily smokers, and 27.5% were occasional smokers. In terms of quantity, more than half of all women who smoked during pregnancy (51.8%) were light smokers, 34.8% were moderate smokers, and 13.5% were heavy smokers [2,3].

Alcohol and smoking during pregnancy are associated with different possible complications: spontaneous abortion, placental abruption, fetal growth restriction, preterm birth, sudden infant death syndrome, and stillbirth [4]. In particular, PAE is considered the leading preventable cause of developmental disabilities and congenital abnormalities [5,6].

Smoking and alcohol induce fetal damage through similar mechanisms, in which oxidative stress appears to play a key role. Indeed, oxidative stress is considered one of the most important mechanisms involved in tobacco smoking during pregnancy [7,8,9,10]. The increase in ROS production from exogenous and endogenous sources results in an imbalance between the generation of oxidant species and antioxidant defenses [11,12,13]. Consequently, ROS in fetal structures may modify the activation of a complex array of genes involved in cell cycle signal transduction and homeostasis control, contributing to defects in endogenous stem cell repair mechanisms [14] and, consequently, the development of several diseases [7,15,16]. The mechanisms through which alcohol inflicts damage on the developing fetus are complex and multifaceted. Alcohol can interfere with cell proliferation, migration, and differentiation, thereby resulting in structural abnormalities in organs and tissues [17]. Ethanol (EtOH) can compromise endogenous antioxidant capacity, for example, by decreasing glutathione peroxidase levels or generating free radicals. Free radicals and ROS, such as superoxide (O^2−^) and hydroxide (HO^−^) ions, are generally considered to be responsible for fetal brain damage by inducing uncontrolled apoptosis [18]. The objective of this study was evaluation of fetal and neonatal effects induced by smoking and alcohol during pregnancy.

## 2. Materials and Methods

We considered at-term pregnancies among those attending the antenatal clinic of the Obstetrics Unit at Sapienza University Rome Policlinico Umberto 1 between 19 April 2023 and 31 May 2024. Exclusion criteria were age <20 or >40 years old, gestational age < 37 weeks + 0 days and >42 weeks + 0 days, multiple pregnancies, placental pathologies, medically assisted procreation, maternal psychiatric, neurological, and/or autoimmune diseases, low socio-economic status, malnutrition, and psychological disorders. Ultrasound was performed to evaluate fetal weight, amniotic fluid index, and maternal-fetal Doppler velocimetry.

Validated questionnaires were used to investigate smoking and alcohol habits. The Fagerstrom Test [19] was chosen to evaluate nicotine dependence related to cigarette smoking. It contains six items that evaluate the quantity of cigarette consumption, the compulsion to use, and dependence. The scores are classified as very low dependence (0–2); low dependence (3,4); medium dependence (5); high dependence (6,7); and very high dependence (8–10). We classified the patient as a passive smoker when the analysis of nicotine was positive while the analysis of cotinine was negative. The Alcohol Use Disorders Identification Test (AUDIT-C) [20] was used to identify patients who may have an active alcohol use disorder. It contains 3 questions; the minimum score (for non-drinkers) is 0, and the maximum possible score is 12. The screening is considered positive if the score is 5 points or greater.

At the time of delivery, we collected the following samples: 1. a tuft of maternal hair (for the analysis of nicotine, cotinine, and ethylglucuronide (EtG)); 2. maternal venous serum blood and cord serum blood (for the analysis of ROS). Placental weight and neonatal data were also collected.

The determination of nicotine, cotinine, and EtG on the maternal keratin matrix was performed using the gas chromatography-mass spectrometry technique.

EtG is a minor non-oxidative metabolite of ethanol, obtained by enzymatic glucuronidation in the liver. Its percentage of formation is less than 1% of the alcohol ingested and reaches maximum concentrations in the blood about 2–3 h after ethanol. EtG is a polar, slightly acidic (pKa = 3.45), and relatively stable molecule and can be detected mainly in urine in a period between 72 and 90 h, but also in blood and the keratin matrix. Blood and urine are used to determine an acute intake of alcohol; on the contrary, the keratin matrix is used to determine the chronic intake.

Nicotine is a toxic parasympathomimetic alkaloid that acts as an agonist for the nicotinic acetylcholine receptor (nAChR), causing the release of dopamine and other neurotransmitters, including norepinephrine, acetylcholine, serotonin, gamma-aminobutyric acid, glutamate, and endorphins. In fact, being a stimulant, it increases the feeling of pleasure and improves mood. Nicotine has two isomers; among these, (S)-nicotine is the most represented in tobacco. Nicotine easily crosses the placental barrier and accumulates in fetal serum and amniotic fluid in concentrations slightly higher than those in maternal serum. Nicotine is extensively metabolized in the liver but also in the lungs and kidneys. The most important metabolite of nicotine is cotinine. Cotinine is used as a biomarker for quantifying exposure to active and passive tobacco smoke. It persists in the body for a long time and can be measured not only in the blood but also in urine, saliva, and hair. Similarly to nicotine, cotinine binds, activates, and desensitizes nicotinic neuronal receptors, although with much lower potency. It has a half-life of about 20 h and is usually detectable for several days, up to a week, after the last use of tobacco.

The quantification of EtG, nicotine, and cotinine is generally performed by gas chromatography (GC) or liquid chromatography (LC), combined with mass spectrometry. In particular, a GC-EI-MS/MS method was applied in this study.

The EtG analysis followed the protocol described by Mattia et al. [21]. Briefly, 50 mg of hair samples were decontaminated by two consecutive washes in 5 mL of methanol and 5 mL of dichloromethane, manually shredded, and incubated overnight at 60 °C in water with EtG-D5 as the internal standard. The extract was purified with solid-phase extraction polymeric cartridges, air-dried, and reconstituted with acetonitrile (CAN) and N-Methyl-N-(trimethylsilyl)trifluoroacetamide (MSTFA) as a derivatizing agent for injection in an Agilent Technologies (Santa Clara, CA, USA) 7890B gas chromatograph coupled to a 7000C tandem mass selective detector operating in EI ionization mode. Hair nicotine and cotinine analyses were performed on an aliquot of 20 mg of hair samples that were decontaminated by sonication for 15 min in 5 mL of dichloromethane, manually shredded, incubated overnight at 60 °C in NaOH 1 M with Nicotine-D4 and Cotinine-D3 as internal standards, and purified with liquid extraction in 1 mL of dichloromethane and 1 mL of 25% K_2_CO_3_. The organic phase was recovered and added with 100 μL of methanol before being concentrated under nitrogen flow to about 100 μL for GC-MS/MS analysis performed by an Agilent Technologies 7890B gas chromatograph coupled to a 7000C tandem mass selective detector operating in EI ionization mode.

A cut-off of 20 pg/mg is the value that allows us to state that the donor of the sample is a habitual drinker. The detection limit for nicotine is 0.16 ng/mg, while for cotinine it is considered a value of 0.07 ng/mg.

The Free Oxygen Radicals Test (FORT) is a colorimetric assay to assess circulating ROS. It is based on the ability of transition metals such as iron to catalyze, in the presence of hydroperoxides (ROOH), the formation of free radicals (reaction 1–2), which are then trapped by an amine derivative, CrNH2 [18]. The amine reacts with free radicals, forming a colored, fairly long-lived radical cation, detectable at 505 nm (reaction 3). The intensity of the color correlates directly to the number of radical compounds and the hydroperoxide concentration and, consequently, to the oxidative status of the sample according to the Lambert–Beer law [18]. According to the instructions provided by the manufacturer, FORT values below 300 units (U) indicate an optimal condition of oxidative stress; for values between 300 and 330 U, a condition of latent oxidative stress; and for values superior to 330 U, a condition of oxidative stress in progress. It should be noted that there are no established reference values for women during delivery and for newborns.

The FORD (Free Oxygen Radical Defense) test is used to evaluate the body’s antioxidant capacity. It uses preformed stable and colored radicals and determines the absorbance decrease proportional to the blood antioxidant concentration of the sample according to Lambert–Beer’s law [18]. In the presence of an acidic buffer (pH = 5.2) and a suitable oxidant (FeCl3), the chromogen (which contains 4-Amino-N, N-diethylaniline sulfate) forms a stable and colored radical cation photometrically detectable at 505 nm. Antioxidant compounds in the sample reduce the chromogen’s radical cation, quenching the color and producing a decoloration of the solution proportional to their concentration. The absorbance values obtained for the samples are compared with a standard curve obtained using Trolox (6-Hydroxy-2,5,7,8-tetramethylchroman-2-carboxylic acid), a permeable cell derivative of vitamin E commonly employed as an antioxidant. FORD results are expressed as Trolox equivalents (mmol/L) using a calibration curve plotted with different amounts of standard Trolox that are stored on the dedicated instrument (FORM Plus, FORM ox, and CR3000 series diagnostic analyzers, Callegari SpA, Catellani Group, Parma, Italy). The test was considered negative (high antioxidant defense capacity) if the value was ≥1.08 Trolox equivalents. 

Statistical analysis: All data are expressed as the mean ± standard deviation (SD) after controlling their normal distribution by the Shapiro–Wilk test. The statistical analysis was performed using Student’s *t*-test for independent samples. The statistical analyses were performed using SPSS software (version 29; IBM Corp., Chicago, IL, USA). A *p*-value of < 0.05 was considered statistically significant.

The study was approved by the ethical committee (protocol number 0382/2023 19 April 2023) and written informed consent was obtained from all subjects involved in the study.

## 3. Results

In this pilot study, we enrolled a total of 119 single pregnant patients. The maternal age range was 21–39 years (mean ± SD: 32.5 years ± 5.73). All enrolled patients were Caucasian. Women’s gestational age at delivery ranged from 37 weeks + 1 day to 41 weeks + 6 days. The mean gestational age at delivery was 39.5 weeks (SD: 2.5). 62% of the patients were nulliparous; the remaining 58% were multiparous. Regarding the mode of delivery, 55% had a spontaneous delivery, while the remaining 45% underwent cesarean section. In 62 out of 119 patients we tested for nicotine and cotinine on the keratin matrix using GC-EI-MS/MS, 26 patients (42%) were active smokers, 18 patients (29%) were passive smokers, and 18 patients (29%) were non-smokers. Analyzing smoking habits, we obtained different results comparing self-reported data (using questionnaires) with measured smoking exposure. In the first case we had 23% of active smokers, while in the second case 42% were active smokers. In the same period, we tested 57 out of 119 pregnant patients for EtG on the keratin matrix. Three patients (5%) out of 57 tested positive for EtG. Analyzing alcohol habits, we had similar results comparing self-reported data (2%) with measured alcohol exposure (5%).

Considering smokers (active smokers *n* = 26 patients, passive smokers *n* = 18 patients) and non-smokers (*n* = 18 patients), we collected the following neonatal data: neonatal weight, APGAR score at the 1st and the 5th minute, umbilical cord pH (UC-pH) level, and placental weight. Average neonatal weights for non-smokers and passive smokers were not different (the 51st pc and the 49th pc, respectively), while average neonatal weight for active smokers was markedly reduced, in the 8th pc. Comparing active smokers and non-smokers, we evidenced a statistically significant difference between the average neonatal weights (*p* = 0.0001) and placental weights (*p* = 0.0060), while the difference between the average pH values was not statistically significant (*p* = 0.5203). Comparing active smokers and passive smokers, we demonstrated a statistically significant difference (*p* = 0.0003) between the average neonatal weights; the difference was not statistically significant between the averages of pH level (*p* = 0.6143) and placental weights (*p* = 0.2492). Analyzing alcohol habits, 3 patients out of 57 tested positive for EtG. Average neonatal weights are in the 1st-2nd pc.

A total of 20 patients (10 active smokers and 10 non-smokers) were tested using the FORT and FORD tests on cord blood and maternal blood (Table 1 and Table 2). The two subgroups were homogeneous in terms of mean age of the patients, parity, gestational age at delivery, mode of delivery, and total number of cigarettes smoked per day. The results on cord blood between active smokers and non-smokers were not statistically significant (FORD test *p* value = 0.2216). Moreover, FORT and FORD test were negative for all newborns, indicating an optimal condition of oxidative stress and an excellent antioxidant status. Comparing FORT results on maternal blood between active smokers and non-smokers, the *p*-value was less than 0.0001, and this difference was considered to be extremely statistically significant. Also comparing FORD test results on maternal blood, the difference was statistically significant (*p* = 0.0156). Given the limited sample size, the results obtained are preliminary and require future studies.

## 4. Discussion

Maternal smoking in pregnancy remains widespread globally in spite of a decline in prevalence due to the public health interventions. In 2020, the global prevalence of smoking during pregnancy was 1.7%, and 250 million women smoke during pregnancy worldwide. The highest prevalence of smoking was observed in Europe at 8.1% [2]. Regarding alcohol consumption during pregnancy, the European Region exhibits the highest prevalence, averaging at 25% [1].

Smoking tobacco and alcohol use during pregnancy may be associated with a number of complications such as abortions, ectopic pregnancy, placental abruption, placenta previa, pre-eclampsia, stillbirth, and sudden infant death syndrome. Preterm birth, smallness-for-gestational age (SGA), fetal growth restriction (FGR), and low birth weight (LBW) are also linked to smoking and alcohol during pregnancy, with established causality [4].

A fetus is considered SGA when individual biometric measurements or a combination of measurements used to estimate fetal weight fall below set parameters and require accurate assessment of gestational age. Commonly, the definition of SGA refers to a fetus with a predicted weight or an abdominal circumference (AC) measurement less than the 10th centile. SGA at birth is commonly diagnosed based on a birthweight below the 10th centile, and often birthweight charts are adjusted for the sex of the baby. FGR implies a pathological restriction of the genetic growth potential. Some, but not all, growth-restricted fetuses/infants have SGA. The likelihood of FGR is higher in fetuses that are smaller. Growth-restricted fetuses may manifest evidence of fetal compromise (abnormal Doppler studies, reduced liquor volume) [22]. LBW is commonly defined as neonatal weight below 2500 g [23].

SGA and FGR are important indicators of increased risk of adverse pregnancy outcome and are associated with perinatal morbidity, neonatal mortality, cerebral palsy, and delayed effects into adolescence and adulthood [24,25,26]. Cigarette smoking during pregnancy is a strong dose-dependent risk factor for SGA and FGR [27]. Smoking alone independently explains 9% and 12% of preterm and term cases of SGA and FGR [28] and has been highlighted as the single largest modifiable risk factor affecting the growth of unborn infants in developed countries [29]. The associations between maternal smoking during pregnancy and the risk of LBW in offspring were reported in population-based observational studies and previous meta-analyses [30,31]. A recent meta-analysis by Hong-Kun Di et al. [32], based on 55 studies including more than 21 million participants from 4 continents, demonstrated that infants whose mothers smoked during pregnancy were 89% more likely to develop LBW, compared with those of non-smoking women. 

The underlying mechanisms of the impact of cigarette smoke on fetal growth and development have been well clarified—hundreds of toxic substances out of more than 7000 chemicals may cross the placental barrier, limit placental development, and restrict fetal growth. Among them, nicotine and carbon monoxide in tobacco smoke were fetal neurotoxins, which could cross the placenta into the fetal circulation, reduce the fetal blood circulation, and subsequently impair fetal oxygen delivery and micronutrients [33]. Moreover, tobacco-specific nitrosamines and polycyclic aromatic hydrocarbons may cause genetic toxicity and carcinogenic and teratogenic effects on the fetus [34].

The mechanisms explaining the relation between PAE and fetal growth are thought to be multifaceted and complex; however, exact molecular targets are unknown [35]. First, it is hypothesized that growth restriction due to PAE arises from induced placental pathology. Alcohol reduces cellular proliferation, affects normal development of the placenta, and potentially results in reduced placental weight and underdeveloped blood vessels, impairing nutrient exchange between mother and fetus [35]. Second, genetic factors might influence growth deficiencies due to PAE. Different studies have reported that 9–14% of children with FASD have chromosomal deletions or duplications, partly explaining FASD features, including growth restriction [35]. Third, the involvement of epigenetic reprogramming and environmental factors, such as nutrition, has been proposed a mechanism influencing fetal growth [35]. Finally, PAE increases oxidative stress. Since in vivo treatment with antioxidants might reduce growth restriction, this is thought to be of influence, although the exact mechanism is not clarified yet [35].

Our data showed a high percentage of smokers during pregnancy: 26 (42%) out of 62 patients were active smokers. Comparing active smokers and non-smokers, mean neonatal weight (8th pc versus 51st pc) and mean placental weight (334 g versus 511 g) were significantly lower for active smokers (*p* = 0.0001 and *p* = 0.0060, respectively). The difference between the average pH values was not statistically significant (*p* = 0.5203). Therefore, the association between maternal smoking during pregnancy and the risk of LBW in offspring was confirmed by our study.

In regard to alcohol, 3 patients out of 57 were positive for EtG. Average neonatal weights were in the 1st-2nd pc, confirming the relation between PAE and fetal growth restriction, despite the limited amount of data available.

Cumulative evidence shows that cigarettes may be the single most significant source of toxic chemical exposure and chemically mediated illness in humans and that maternal smoke exposure in pregnancy can cause substantial harm to women and their developing fetuses. Nicotine readily crosses the placental barrier and has been found in the amniotic fluid and umbilical cord of neonates [36].

It has become evident that oxidative stress is one of the most important mechanisms involved in tobacco smoking during pregnancy [37]. The increase in ROS production from exogenous and endogenous sources results in an imbalance between the generation of oxidant species and antioxidant defenses. Consequently, ROS in fetal structures may modify the activation of a complex array of genes involved in cell cycle signal transduction and homeostasis control, contributing to defects in endogenous stem cell repair mechanisms and, consequently, the development of many diseases [16]. One possibility given credence by several in vitro studies is that cigarette smoke, rich in free radicals and oxidizing species, depletes plasma antioxidants [38]. Cigarette smoking causes oxidative stress in pregnant women and may have a similar effect in fetuses.

As we have already explained for cigarette smoking, EtOH can also compromise endogenous antioxidant capacity, for example, by decreasing glutathione peroxidase levels or generating free radicals. Free radicals and ROS, such as superoxide (O_2_^−^) and hydroxide (HO^−^) ions, are generally considered to be responsible for fetal brain damage by inducing uncontrolled apoptosis [18].

Given the small number of patients who tested positive for alcohol consumption during pregnancy, we focused our attention on oxidative stress in smoking patients.

Ednildes de Almeida Olympio Rua et al. [39] evaluated the impact of smoking during gestation on the viability of blood mononuclear cells (MNC) from umbilical cords of newborns. Pregnant smokers had a reduction in MNC viability from the umbilical cord (10%), an increase in the production of ROS, and an increase in cell apoptosis compared to pregnant non-smokers [39]. These data show that maternal cigarette smoking during pregnancy may compromise the viability of MNC and damage the umbilical cord structure, possibly by excessive ROS bioavailability [39].

Ali Aycicek [40] evaluated the influence of active and passive maternal smoking on cord blood total oxidant/antioxidant status at term, including 29 non-smokers, 30 passive smokers, and 21 active smokers. The gestation period of all pregnancies was between 37 and 40 weeks, the pregnancies were uncomplicated, and the infants were delivered vaginally. Significantly lower concentrations of catalase (CAT), paraoxonase 1 (PON1), and total antioxidant capacity (TAC) were found in the cord blood of the smokers than in that of the non-smokers (*p* < 0.018). The cord blood levels of thiol and lipid hydroperoxide (LOOH) and total oxidant status (TOS) and oxidative stress index (OSI) were significantly higher in the active and passive smokers than in the controls (*p* < 0.01). A significant positive correlation was found between maternal tobacco exposure and cord blood OSI (*p* < 0.001). The authors demonstrated that active or passive maternal smoking was associated with important alterations in the oxidant and antioxidant balance in fetal cord blood, causing potent oxidative stress. Also, Chelchowska M et al. [41] and Kurt A. et al. [42] reported that the level of TAC was significantly decreased in the cord blood of newborns of smoking mothers. Chelchowska M et al. also reported in another study that the concentration of malondialdehyde (MDA) in the plasma of cord blood of newborns of smoking mothers was significantly higher (*p* < 0.01), but the antioxidant defense was lower (*p* < 0.0001) than in non-smoking ones [43]. 

On the other hand, Fayol et al. [44] demonstrated that the TAC level was low in the infant cord blood of passive smoking mothers but that this was not the case in the infants of active smokers, hypothesizing an interesting defense mechanism in the fetus of active smoking patients.

Our study, considering two homogeneous subgroups (*n* = 10 non-smokers and *n* = 10 active smokers), showed a statistically significant higher oxidative stress in the blood of active smoking patients (FORT *p* < 0.0001; FORD *p* = 0.0156). In cord blood the differences were not statistically significant; in particular, FORT and FORD tests were negative in all cases, indicating a condition of oxidative defense even in fetuses of active smoking mothers. This result is in agreement with the results by Fayol et al. [44]. Given the limited sample size, the results obtained are preliminary and require future studies.

Limitations of the study: the small sample size for determination of oxidative stress and the single-center design. A future goal is to increase the sample size, also for drinking patients, to provide greater robustness to the results.

## 5. Conclusions

Our study aims to emphasize the importance of providing pregnant women with accurate information regarding the potential risks and complications associated with exposure to tobacco and alcohol during pregnancy. Fetal growth restriction related to smoking and alcohol exposure is preventable. Smoking induces oxidative stress in pregnant women, while the fetus appears to experience a state of oxidative defense, likely induced by the mother, the mechanisms of which are not yet fully understood. It is important to conduct further studies in the future to explore these mechanisms.

## Figures and Tables

**Table 1:** FORT and FORD test results for active smoker patients and their children.

	CORD BLOOD		MATERNAL BLOOD	
ID	FORT (U)	FORD (mmol/L di Trolox)	FORT (U)	FORD (mmol/L di Trolox)
**07nic**	150	1.65	648	0.83
**22nic**	150	1.49	599	0.80
**24nic**	150	1.77	486	0.93
**25nic**	150	1.70	556	1.00
**30nic**	150	1.58	517	0.87
**42nic**	150	1.31	532	0.85
**53nic**	150	1.46	517	0.87
**56nic**	150	1.67	368	1.20
**58nic**	150	1.56	553	0.90
**61nic**	150	1.61	548	0.91
**Mean**	150	1.58	532.4	0.916
**SD**	0	0.1334	73.54	0.1143

**Table 2:** FORT and FORD test results for non-smoker patients and their children.

	CORD BLOOD		MATERNAL BLOOD	
ID	FORT (U)	FORD (mmol/L di Trolox)	FORT (U)	FORD (mmol/L di Trolox)
**02nic**	150	1.62	315	1.03
**04nic**	150	1.57	350	1.00
**10nic**	150	1.68	338	0.98
**15nic**	150	1.69	439	1.09
**21nic**	150	1.58	367	1.10
**32nic**	150	1.87	310	0.99
**35nic**	150	1.55	325	0.95
**41nic**	150	1.51	339	1.00
**45nic**	150	1.77	329	0.98
**50nic**	150	1.65	308	1.20
**Mean**	150	1.649	342	1.032
**SD**	0	0.1091	38.72	0.0761

## Data Availability

The original contributions presented in this study are included in the article. The data collected from patients are stored anonymously in databases. Further inquiries can be directed to the corresponding author.
